# Significance of Genital Mycoplasmas in Pelvic Inflammatory Disease: Innocent Bystander!

**DOI:** 10.1155/S1064744996000518

**Published:** 1996

**Authors:** Ashwin Chatwani, Ozgur H. Harmanli, Paul Nyirjesy, E. Albert Reece

**Affiliations:** Department of Obstetrics, Gynecology, and Reproductive Sciences Temple University School of Medicine 3401 N. Broad Street Philadelphia PA 19140 USA

## Abstract

*Objective:* Our objective was to determine the role of *Mycoplasma hominis* and *Ureaplasma urealyticum* in pelvic inflammatory disease (PID).

*Methods:* The clinical and microbiologic variables in 114 patients with a clinical diagnosis of PID were compared prospectively according to the isolation of *M. hominis* and *U. urealyticum* from their endometrial cavities.

*Results:* The groups were epidemiologically well matched. Clinical parameters such as temperature, leukocyte count, erythrocyte count, and C-reactive protein on admission and length of hospital stay were similar in the patients, regardless of their mycoplasma status. A significant percentage of the patients either continued or started to harbor genital mycoplasmas after the resolution of PID without any significant clinical sequelae.

*Conclusions:* The presence of genital mycoplasmas does not change the clinical presentation and course of PID. Both *M. hominis* and *U. urealyticum* can persist or colonize the endometrium after complete recovery from PID. Therefore, the genital mycoplasmas do not seem to have a dominant pathogenic role in PID.


Infectious Diseases in Obstetrics and Gynecology 4:263–268 (1996)
© 1997 Wiley-Liss, Inc.

### Significance of Genital Mycoplasmas in Pelvic Inflammatory Disease: Innocent Bystander!

**Ashwin Chatwani,* Ozgur H. Harmanli, Paul Nyirjesy, and E. Albert Reece**

*Department of Obstetrics, Gynecology, and Reproductive Sciences, Temple University School of Medicine, Philadelphia, Pennsylvania*

### Abstract

***Objective:*** **Our objective was to determine the role of *Mycoplasma hominis* and *Ureaplasma urealyticum* in pelvic inflammatory disease (PID).**

***Methods:*** **The clinical and microbiologic variables in 114 patients with a clinical diagnosis of PID were compared prospectively according to the isolation of *M. hominis* and *U. urealyticum* from their endometrial cavities.**

***Results:*** **The groups were epidemiologically well matched. Clinical parameters such as temperature, leukocyte count, erythrocyte count, and C-reactive protein on admission and length of hospital stay were similar in the patients, regardless of their mycoplasma status. A significant percentage of the patients either continued or started to harbor genital mycoplasmas after the resolution of PID without any significant clinical sequelae.**

***Conclusions:*** **The presence of genital mycoplasmas does not change the clinical presentation and course of PID. Both *M. hominis* and *U. urealyticum* can persist or colonize the endometrium after complete recovery from PID. Therefore, the genital mycoplasmas do not seem to have a dominant pathogenic role in PID. Infect. Dis. Obstet. Gynecol. 4:263–268, 1996.** © 1997 Wiley-Liss, Inc.

Key words genital mycoplasmas; pelvic inflammatory disease; *Mycoplasma hominis; Ureaplasma urealyticum*

Mycoplasmas have been recognized as pathogens in a number of animals.[1] Since the initial isolation of mycoplasma in a culture from a Bartholin's gland abscess in 1937, mycoplasmas have been implicated in a variety of human diseases.[2] Their etiologic role in human atypical pneumonia is well established.[1] Despite some earlier data suggesting an association between mycoplasmas and pelvic inflammatory disease (PID), the role of these organisms in upper-genital-tract infections remains controversial.

Three different mycoplasma species have been isolated from the human genital tract: *Mycoplasma fermentans, Mycoplasma hominis,* and *Ureaplasma urealyticum.* The latter 2 have been linked to PID and other gynecologic or reproductive-tract disorders.[1]

In this study, our objective was to determine the role of *M. hominis* and *U. urealyticum* in PID. For this purpose, we studied patients with positive endometrial cultures for *M. hominis* or *U. urealyticum* to assess the effects of these organisms on the clinical presentation and course of PID; their association with other microorganisms; and their presence after the resolution of PID.

### MATERIALS AND METHODS

The subjects for this study represented patients participating in 2 large controlled, prospective an-

*Correspondence to: Dr. Ashwin Chatwani, Department of Obstetrics, Gynecology, and Reproductive Sciences, Temple University School of Medicine, 3401 N. Broad Street, Philadelphia, PA 19140.

Received 15 May 1996

Accepted 17 September 1996

tibiotic trials. The protocols of these studies were approved by the Institutional Review Board of Temple University Hospital. After giving signed informed consents, 114 consecutive patients with a clinical diagnosis of acute PID were enrolled. The diagnosis of PID was based on the presence of abdominal pain and tenderness, cervical motion tenderness, and adnexal tenderness plus at least one of the following findings: elevated temperature (>38°C, 100.4°F), leukocytosis (>10,000/mm$^3$), elevated erythrocyte sedimentation rate (>20 mm/h), elevated C-reactive protein (>0.0625 mg/dl), or a purulent vaginal discharge.

Cervical cultures were obtained for *Neisseria gonorrhoeae* and *Chlamydia trachomatis*. After cleansing the cervix with a povidone-iodine antiseptic solution, we obtained an endometrial aspirate with an endometrial aspiration curette (Pipelle™, Unimar, Inc., Wilton, CT) and cultures for *M. hominis*, *U. urealyticum*, *N. gonorrhoeae*, *C. trachomatis*, anaerobes, and aerobes. Other laboratory studies included leukocyte count, erythrocyte sedimentation rate, and C-reactive protein.

The samples for genital mycoplasmas were placed into Shephard's 10B transport broth and transferred to the reference laboratory at –70°C on dry ice.[1] The specimens were inoculated into arginine broth and Ford's broth for *M. hominis* and *U. urealyticum*, respectively. The growth of mycoplasmas was suggested by an alkaline pH shift due to arginine hydrolysis by *M. hominis* and urease activity by *U. urealyticum*. Cultures in $CO_2$ on A-7 agar were used for further differentiation and confirmation of the morphology.[3]

The patients were admitted to the hospital and treated with antibiotics. The investigational antibiotics to which *M. hominis* and *U. urealyticum* were sensitive were used. The patients were monitored daily by measurements of their vital signs and physical examinations. The laboratory tests, specifically WBC count, C-reactive protein, and sedimentation rate, were repeated every 3rd day while the patient was hospitalized. The patient was discharged home on antibiotics after resolution of the signs and symptoms of PID. The laboratory tests were repeated at the time of discharge. Eighty-eight patients returned for follow-up visits. The cultures were repeated 5–7 days and 21–28 days after the completion of the outpatient treatment.

TABLE 1A. *M. hominis* in endometrial cultures and epidemiologic variables[a]

| | *M. hominis* present (N = 81) | *M. hominis* absent (N = 33) | *P* |
|---|---|---|---|
| Age (years) | 24.9 ± 5.8 | 26.3 ± 6.4 | NS |
| History of STD | 38 (46.5%) | 15 (45.5%) | NS |
| History of PID | 13 (16.5%) | 5 (15.2%) | NS |

[a]Age is given as mean ± standard deviation. NS, not significant.

TABLE 1B. *U. urealyticum* in endometrial cultures and epidemiologic variables[a]

| | *U. urealyticum* present (N = 44) | *U. urealyticum* absent (N = 70) | *P* |
|---|---|---|---|
| Age (years) | 24.7 ± 6.5 | 25.7 ± 5.6 | NS |
| History of STD | 20 (45.5%) | 33 (47.1%) | NS |
| History of PID | 6 (13.6%) | 12 (17.1%) | NS |

[a]Age is given as mean ± standard deviation. NS, not significant.

TABLE 1C. *M. hominis* plus *U. urealyticum* and epidemiologic variables[a]

| | Both present (N = 33) | Both absent (N = 81) | *P* |
|---|---|---|---|
| Age (years) | 24.5 ± 6.0 | 25.7 ± 6.0 | NS |
| History of STD | 17 (51.2%) | 36 (44.4%) | NS |
| History of PID | 6 (18.2%) | 12 (14.8%) | NS |

[a]Age is given as mean ± standard deviation. NS, not significant.

The laboratory tests were repeated only at the first follow-up.

Statistical analyses were performed using the Mantel-Haenszel chi-squared formula. When a cell value of <5 was encountered, a 2-tailed *P* value was obtained by means of the Fisher's exact test. For continuous variables, a *P* value was calculated through a 1-way analysis of the means. $P < 0.05$ was considered significant.

### RESULTS

The groups with and without positive endometrial cultures for genital mycoplasmas were well matched for age, histories of PID, and sexually transmitted diseases, (STDs) (Tables 1A–C).

The mean temperature, leukocyte count, and C-reactive protein values on admission and the

TABLE 2A. *M. hominis* in endometrial cultures and clinical parameters[a]

| | *M. hominis* present (N = 81) | *M. hominis* absent (N = 33) | *P* |
|---|---|---|---|
| WBC count (/mm$^3$) | 16.7 ± 5.5 | 15.5 ± 5.6 | NS |
| Temperature, °C (°F) | 38.22 ± 2.7 (100.8 ± 1.5) | 38.27 ± 2.2 (100.9 ± 1.2) | NS |
| C-reactive protein (ng/ml) | 9.5 ± 7.0 | 7.5 ± 6.3 | NS |
| Length of stay (days) | 4.1 ± 0.9 | 4.3 ± 1.2 | NS |

[a]All values are given as mean ± standard deviation. NS, not significant.

TABLE 2B. *U. urealyticum* in endometrial cultures and clinical parameters[a]

| | *U. urealyticum* present (N = 44) | *U. urealyticum* absent (N = 70) | *P* |
|---|---|---|---|
| WBC count (/mm$^3$) | 16.7 ± 5.6 | 16.1 ± 5.5 | NS |
| Temperature, °C (°F) | 38.38 ± 2.7 (101.1 ± 1.5) | 38.16 ± 2.3 (100.7 ± 1.3) | NS |
| C-reactive protein (ng/ml) | 9.3 ± 8.0 | 8.9 ± 7.4 | NS |
| Length of stay (days) | 4.3 ± 1.2 | 4.1 ± 0.9 | NS |

[a]All values are given as mean ± standard deviation. NS, not significant.

length of hospital stay according to the presence of genital mycoplasmas in the endometrial cultures were not significant (Tables 2A–C).

No statistically significant difference in isolation rates of anaerobes, aerobes, gonococci, or chlamydiae was found between the patients with positive and negative endometrial mycoplasmas (Tables 3A–C).

On admission, 81 of 114 patients (71.2%) had positive endometrial cultures for *M. hominis* (Table 4). Although there was a significant decrease in recovery rate of this organism after the complete resolution of the symptoms and signs of PID ($P < 0.05$), a significant percentage of patients continued to be positive for *M. hominis* at the first (28.4%) and second (29.5%) follow-up visits.

*U. urealyticum* was isolated from the endometrial specimens of 44 of 114 patients (38.6%) on admission. The isolation rates continued to be significant at the first (21.6%) and second (19.3%) follow-up visits (Table 4). This trend was observed in the isolation rates of both mycoplasmas (Table 4).

Of those patients who were culture-positive for *M. hominis* upon admission, 37.1% and 40.3% were still positive for this organism at the first and second follow-up visits, respectively (Table 5A). In contrast, *U. urealyticum* was recovered from these follow-up endometrial cultures in only a minority (15.2% and 18.2%, respectively) of the patients who had this organism on admission (Table 5B). Interestingly, some patients who initially had negative endometrial cultures for genital mycoplasmas became positive for these organisms at their follow-up examinations: 7.7% for *M. hominis*, and 15.5% for *U. urealyticum* at the first follow-up visit and

TABLE 2C. Both *M. hominis* and *U. urealyticum* in endometrial cultures and clinical parameters[a]

| | Both present (N = 33) | Both absent (N = 81) | *P* |
|---|---|---|---|
| WBC count (/mm$^3$) | 16.9 ± 4.9 | 16.1 ± 5.8 | NS |
| Temperature, °C (°F) | 38.55 ± 29 (101.4 ± 1.6) | 38.22 ± 2.3 (100.8 ± 1.3) | NS |
| C-reactive protein (ng/ml) | 10.1 ± 8.2 | 8.6 ± 7.3 | NS |
| Length of stay (days) | 4.2 ± 1.2 | 4.2 ± 1.0 | NS |

[a]All values are given as mean ± standard deviation. NS, not significant.

3.8% for *M. hominis* and 20.0% for *U. urealyticum* at the second follow-up visit (Tables 5A,B).

There were 5 failures in this study. One patient positive for both *M. hominis* and *U. urealyticum* required surgery for bilateral tubo-ovarian abscessed. Two patients with positive *M. hominis* and 2 patients with negative cultures for mycoplasmas required changes of antibiotics because of failure to respond to the original antibiotics. All patients who returned for follow-up visits complied with the protocols by abstaining from intercourse during the study period.

### DISCUSSION

The significance of mycoplasmas in PID has been extensively studied. However, controversy over the significance and pathogenicity of genital mycoplasmas in pelvic infections continues. In a group of 50 women with laparoscopically verified acute salpin-

TABLE 3A. Recovery of other microorganisms from endometrial cultures of patients with *M. hominis*[a]

| | *M. hominis* present (N = 81) | *M. hominis* absent (N = 33) | *P* |
|---|---|---|---|
| Anaerobes | 34 (42.0%) | 11 (33.3%) | NS |
| Aerobes | 46 (56.8%) | 17 (51.2%) | NS |
| *N. gonorrhoeae* | 51 (62.3%) | 17 (51.2%) | NS |
| *C. trachomatis* | 16 (19.8%) | 6 (18.2%) | NS |

[a]NS, not significant.

TABLE 3B. Recovery of other microorganisms from endometrial cultures of patients with *U. urealyticum*[a]

| | *U. urealyticum* present (N = 44) | *U. urealyticum* absent (N = 70) | *P* |
|---|---|---|---|
| Anaerobes | 18 (40.9%) | 27 (38.6%) | NS |
| Aerobes | 26 (59.1%) | 37 (52.9%) | NS |
| *N. gonorrhoeae* | 24 (54.5%) | 44 (62.9%) | NS |
| *C. trachomatis* | 11 (25.0%) | 11 (15.7%) | NS |

[a]NS, not significant.

TABLE 3C. Recovery of other microorganisms from endometrial cultures of patients with both *M. hominis* and *U. urealyticum*[a]

| | Both present (N = 33) | Both absent (N = 81) | *P* |
|---|---|---|---|
| Anaerobes | 13 (39.4%) | 32 (39.5%) | NS |
| Aerobes | 21 (63.6%) | 12 (14.8%) | NS |
| *N. gonorrhoeae* | 20 (60.6%) | 48 (59.3%) | NS |
| *C. trachomatis* | 9 (27.3%) | 13 (16.1%) | NS |

[a]NS, not significant.

TABLE 4. Persistence of mycoplasmas in endometrial cultures at follow-up visits

| | *M. hominis* present | *U. urealyticum* present | Both *M. hominis* and *U. urealyticum* present |
|---|---|---|---|
| On admission (N = 114) | 81 (71.2%) | 44 (38.6%) | 33 (28.9%) |
| 1st follow-up (N = 88) | 25 (28.4%) | 19 (21.6%) | 6 (20.1%) |
| 2nd follow-up (N = 88) | 26 (29.5%) | 17 (19.3%) | 5 (18.3%) |

gitis, Mardh and Westrom[4] showed *M. hominis* and *U. urealyticum* in the fallopian-tube specimens of 4 (8%) and 2 (4%) of the patients, respectively, whereas none of the 34 controls had mycoplasma cultured from their tubes. Of note, none of them

TABLE 5A. *M. hominis* in endometrical cultures at follow-up visits

| | *M. hominis* present on admission (N = 62) | *M. hominis* absent on admission (N = 26) |
|---|---|---|
| *M. hominis* present at 1st follow-up | 23 (37.1%) | 2 (7.7%) |
| *M. hominis* present at 2nd follow-up | 25 (40.3%) | 1 (3.8%) |

TABLE 5B. *U. urealyticum* in endometrial cultures at follow-up visits

| | *U. urealyticum* present on admission (N = 33) | *U. urealyticum* absent on admission (N = 55) |
|---|---|---|
| *U. urealyticum* present at 1st follow-up | 5 (15.2%) | 14 (15.5%) |
| *U. urealyticum* present at 2nd follow-up | 6 (18.2%) | 11 (20.0%) |

had positive tubal cultures for *N. gonorrhoeae*. Other investigators were also able to isolate *M. hominis* or *U. urealyticum* from pelvic abscesses and tubal and cul-de-sac specimens of PID patients, either laparoscopically or through culdocentesis, in similar rates.[5,6] Moller et al.[7] demonstrated the development of PID after hysterosalpingography in 2 patients with positive cervical cultures for *M. hominis*, 1 of whom also showed a statistically significant rise in the titer of antibodies against this organism. In another report by the same investigators,[8] the evidence of pelvic inflammation was revealed in the form of parametritis rather than salpingitis after the inoculation of *M. hominis* directly into the fallopian tubes of grivet monkeys. Mardh and coworkers,[9] employing electron microscopy, showed swelling of the fallopian-tube cilia secondary to *M. hominis* in organ culture systems. The role of mycoplasmas in PID was also supported by serologic studies that showed the presence and elevation of *M. hominis* antibodies in 25–40% of patients with PID.[9–11]

In contrast, Sweet and Gibbs[10] and Eschenbach and colleagues[12] found no difference in cervical colonization of mycoplasmas in patients with or without PID. Sweet et al.[6] were not able to recover *M. hominis* in fallopian-tube specimens of 39 patients with laparoscopically confirmed PID, although 80% of these patients had positive cervical cultures for *M. hominis*. Using fallopian-tube organ cultures, Taylor-Robinson and Carney[13] noted no tissue damage with *M. hominis* inoculation despite the extensive epithelial damage caused by *N. gonorrhoeae.* Gump et al.[14] were able to culture mycoplasma from 10 of 203 (4.9%) endometrial biopsy specimens that showed no histologic signs of inflammation. A number of other researchers[15,16] were also unable to report any association between genital mycoplasmas and PID.

In this study, we compared the clinical and microbiologic variables in patients with a clinical diagnosis of PID according to the recovery of genital mycoplasmas from their endometrial cavities. It has been proposed that the best way to identify the microorganisms involved in PID is through culture of the fallopian-tube exudate. However, this technique is invasive as it involves laparoscopy. It has been demonstrated that isolates obtained from the endometrial cavities mirror those in the fallopian tubes more closely than those obtained by other means, including culdocentesis.[6] Therefore, we believe that the endometrial culture results presented in this study reflect a spectrum of microorganisms similar to that present in the fallopian tubes.

Our results provide evidence of a poor association between acute PID and genital mycoplasmas. Our findings indicate that the presence of genital mycoplasmas does not change the clinical presentation or the clinical course of PID. The clinical parameters such as temperature, leukocyte count, erythrocyte count, and C-reactive protein on admission were similar in our patients, regardless of their mycoplasma statuses. Furthermore, the patients with and without genital mycoplasmas in their upper genital tracts were discharged in the same period of time after becoming free of the symptoms and signs of PID. A significant number of patients continued to be positive for mycoplasmas even after resolution of the symptoms and signs of PID and normal leukocyte counts, C-reactive protein, and sedimentation rates.

Our study is the first in which patients with PID have been prospectively followed and serial cultures of the upper genital tract for mycoplasmas have been obtained. By using these patients as their own controls, we were able to demonstrate the persistence of mycoplasmas in the endometrial cultures of women who no longer had the symptoms and signs of PID. The follow-up cultures showed that asymptomatic women can be positive for *M. hominis* or *U. urealyticum* in their upper genital tracts. This finding may be explained either by persistence of genital mycoplasmas in the endometrial cavity after complete clinical recovery of acute PID or by colonization of the endometrium by these organisms.

Based on our data, we postulate that genital mycoplasmas have a secondary pathogenic role if any, in acute PID. It is also possible that genital mycoplasmas colonize the endometrium.

### REFERENCES

1. Valleca WM, Bird BR: Laboratory diagnosis of mycoplasma infections. Course 8226-C. Washington, DC: U.S. Department of Health and Human Services, Public Health Services Center for Disease Control, October 1980.
2. Dienes L, Edsall J: Observation on the L organism of Kleineberger. Proc Soc Exp Biol Med 36:740–744, 1937.
3. Freundt EA: Culture media for classical mycoplasmas. In Tully JG, Razin S (eds): Methods in Mycoplasmology. Vol 1. New York: Academic Press, 1983.
4. Mardh P-A, Westrom L: Tubal and cervical cultures in acute salpingitis with special reference to *Mycoplasma hominis* and T-strain mycoplasmas. Br J Vener Dis 46: 179–186, 1970.
5. Friberg J: Genital mycoplasma infections. Am J Obstet Gynecol 132:573–578, 1978.
6. Sweet RL, Draper DL, Schachter J, et al.: Microbiology and pathogenesis of acute salpingitis as determined by laparoscopy: What is the appropriate site of sample? Am J Obstet Gynecol 138:985–989, 1980.
7. Moller BR, Allen J, Toft B, et al.: Pelvic inflammatory disease after hysterosalpingography associated with *Chlamydia trachomatis* and *Mycoplasma hominis.* Br J Obstet Gynaecol 91:1181–1187, 1984.
8. Moller BR, Freundt EA, Mardh P-A: Experimental pelvic inflammatory disease provoked by *Chlamydia trachomatis* and *Mycoplasma hominis* in grivet monkeys. Am J Obstet Gynecol 138:990–995, 1980.
9. Mardh P-A, Westrom L, van Mecklenberg C, et al.: Studies on ciliated epithelia of the human genital tract. I. Swelling of the cilia of fallopian tube epithelium in organ cultures infected with *Mycoplasma hominis.* Br J Vener Dis 52:52–57, 1976.
10. Sweet RL, Gibbs RS: Pelvic inflammatory disease. In Sweet RL, Gibbs RS (eds): Infectious Diseases of the Female Genital Tract. Baltimore: Williams & Wilkins, pp 53–77, 1985.
11. Moller BR: The role of mycoplasmas in the upper genital tract of women. Sex Transm Dis 10(Suppl):281–284, 1983.

12. Eschenbach DA, Buchanan T, Pollock HM, et al.: Polymicrobial etiology of acute pelvic inflammatory disease. N Engl J Med 293:166–171, 1975.
13. Taylor-Robinson D, Carney FE: Growth and effect of mycoplasmas in fallopian tube organ cultures. Br J Vener Dis 50:212–216, 1974.
14. Gump DI, Gibson M, Ashikaga T: Lack of association between genital mycoplasmas and infertility. N Engl J Med 310:937–941, 1984.
15. Henry-Suchet J, Catalan F, Loffredo V, et al.: *Chlamydia trachomatis* associated with chronic inflammation in abdominal specimens from women selected for tuboplasty. Fertil Steril 36:599–605, 1981.
16. Cassell GH, Younger JB, Brown MB, et al.: Microbiologic study of infertile women at the time of diagnostic laparoscopy: Association with *Ureaplasma urealyticum* with a defined subpopulation. N Engl J Med 308:502–505, 1983.
